# Signature construction and molecular subtype identification based on cuproptosis-related genes to predict the prognosis and immune activity of patients with hepatocellular carcinoma

**DOI:** 10.3389/fimmu.2022.990790

**Published:** 2022-09-28

**Authors:** Xingyu Peng, Jinfeng Zhu, Sicheng Liu, Chen Luo, Xun Wu, Zitao Liu, Yanzhen Li, Rongfa Yuan

**Affiliations:** ^1^ Department of General Surgery, The Second Affiliated Hospital of Nanchang University, Nanchang, China; ^2^ Jiangxi Province Key Laboratory of Molecular Medicine, The Second Affiliated Hospital of Nanchang University, Nanchang, China; ^3^ Department of General Surgery, The Second Xiangya Hospital of Central South University, Changsha, China; ^4^ Department of Clinical Medicine, Nanchang Medical College, Nanchang, China

**Keywords:** cuproptosis, hepatocellular carcinoma, immune infiltration, prognostic signature, immune microenvironment

## Abstract

**Background:**

Hepatocellular carcinoma (HCC) is one of the most common malignancies in the world, with high incidence, high malignancy, and low survival rate. Cuproptosis is a novel form of cell death mediated by lipoylated TCA cycle proteins-mediated novel cell death pathway and is highly associated with mitochondrial metabolism. However, the relationship between the expression level of cuproptosis-related genes (CRGs) and the prognosis of HCC is still unclear.

**Methods:**

Combining the HCC transcriptomic data from The Cancer Genome Atlas(TCGA) and Gene Expression Omnibus (GEO) databases, we identified the differentially expressed cuproptosis-related genes (DECRGs) and obtained the prognosis-related DECRGs through univariate regression analysis.LASSO and multivariate COX regression analyses of these DECRGs yielded four genes that were used to construct the signature. Next, we use ROC curves to evaluate the performance of signatures. The tumor microenvironment, immune infiltration, tumor mutation load, half-maximum suppression concentration, and immunotherapy effects were also compared between the low-risk and high-risk groups. Finally, we analyzed the expression level, prognosis, and immune infiltration correlation on the four genes that constructed the model.

**Results:**

Four DECRGs s were used to construct the signature. The ROC curves indicated that signature can better assess the prognosis of HCC patients. Patients were grouped according to the signature risk score. Patients in the low-risk group had a significantly longer survival time than those in the high-risk group. Furthermore, the tumor mutation burden (TMB) values were associated with the risk score and the higher-risk group had a higher proportion of TP53 mutations than the low-risk group.ESTIMATE analysis showed significant differences in stromal scores between the two groups.N6-methyladenosine (m6A) and multiple immune checkpoints were expressed at higher levels in the high-risk group. Then, we found that signature score correlated with chemotherapeutic drug sensitivity and immunotherapy efficacy in HCC patients. Finally, we further confirmed that the four DECRGs genes were associated with the prognosis of HCC through external validation.

**Conclusions:**

We studied from the cuproptosis perspective and developed a new prognostic feature to predict the prognosis of HCC patients. This signature with good performance will help physicians to evaluate the overall prognosis of patients and may provide new ideas for clinical decision-making and treatment strategies.

## Introduction

Liver cancer is a malignant disease of the digestive system and ranks the third cause of cancer-related deaths worldwide (1). Only a small proportion of patients with early liver cancer can be cured by surgical resection (2). Hepatocellular carcinoma (HCC) is the majority of primary liver cancer, with up to 850,000 new cases occurring each year (3). Although it has been shown that the main risk factors for HCC are associated with a sustained virological response to hepatitis C, hepatitis B virus suppression in treatment, and alcoholic and nonalcoholic fatty liver disease (4). But its etiology and molecular mechanisms remain largely unknown (5).HCC is a highly heterogeneous disease, with intratumoral morphological and genetic heterogeneity further complicating our understanding of hepatocarcinogenesis (6).

Copper is an essential nutrient involved in various biological functions, and its redox properties make it both beneficial and toxic to cells (7). The imbalance of copper can cause oxidative stress in the body and thus affect tumor development (8). Copper has recently been found to induce cell death by targeting lipoylated TCA cycle proteins (9). Cuproptosis is a new form of programmed cell death (10), which is different from the cell death associated with oxidative stress (such as cell apoptosis (11), ferroptosis (12), and necroptosis (13). The finding of cuproptosis reinforces the idea that mitochondria are multifaceted regulators of cell death (14)and also challenges the conventional idea that oxidative stress is the fundamental molecular mechanism of metal-induced toxicity (15). Some recent reports suggest that mitochondria can affect drug resistance in cancer, leading to poor chemical therapy effects in HCC patients (16, 17). Zhang et al. (18) showed that copper content is closely related to hepatocellular carcinoma (HCC), and that serum copper and ceruloplasmin levels can be used as markers to detect HCC. In addition, the study by Koizumi et al. (19) demonstrated that elevated levels of redox-active free copper are closely associated with HCC due to acute hepatitis. Additionally, Siddiqui et al. (20)showed that CuO NPs can induce apoptosis in human hepatocellular carcinoma (HepG2) cells *via* ROS through the mitochondrial pathway. The above studies show that copper plays an important role in the development of HCC, indicating that cuproptosis may be closely related to the development of HCC. However, whether cuproptosis is related to the prognosis of liver cancer patients has not been studied.

Tumor mutational burden (TMB) is the number of somatic mutations per megabase of the interrogated genome sequence in a tumor sample, with the potential for predictive biomarkers (21).TMB plays an important role in the immunotherapy of tumors, and the higher the TMB, the better the immunotherapy benefits (22, 23). It has been shown that non-small-cell lung cancer and colorectal cancer with high TMB values may have a poor prognosis (24, 25). It has been shown that high TMB in HCC patients has a worse prognosis than patients with low TMB (26). However, it has also been suggested that higher TMB levels indicate longer overall survival (27). Therefore, whether TMB can be used as a biomarker for HCC remains unclear.

To explore the prognostic value of cuproptosis-related genes (CRGs) and the relationship with tumor mutations and immunotherapy, differential expression was performed by analysis and prognostic analysis of CRGs. We then constructed a new prognostic gene signature using four differentially expressed cuproptosis-related genes (DECRGs). Our data suggest that risk scores and staging were identified as independent prognostic factors. Furthermore, we explored the impact of risk scores on TMB and immunotherapy to further assess the value of signature in molecular therapy. Finally, we performed external validation of the expression levels and prognostic value of the four genes in the signature.

## Materials and methods

### Multiomics data collection and processing

In the first, we downloaded the gene transcriptome data (n = 424), clinical data (n = 377), and gene mutation data (n = 364) of patients with HCC from The Cancer Genome Atlas (TCGA) database (https://portal.gdc.cancer.gov/). Fragments per Kilobase million were used for the transcriptome data, which subsequently transformed into transcripts per million (TPM). We processed the survival information of HCC patients and deleted one sample with incomplete survival information. Next, we downloaded the GSE76427 dataset from the Gene Expression Omnibus (GEO) database (https://www.ncbi.nlm.nih.gov/geo/) and retained the tumor sample information for merging with the TCGA data. The clinical characteristics of all HCC patients are shown in **Table 1**. Digital focal-level copy number variation (CNV) was downloaded from the GDC TCGA Liver Cancer (LIHC) project on the UCSC Xena server(https://xena.ucsc.edu/).

**Table 1:** The clinical characteristics of the TCGA cohort and GSE76427 cohort.

Variables	TCGA cohort (N = 376)	GSE76427 cohort (N = 115)
Age		
≤ 65 years	235	65
>65 years	141	50
Sex		
Female	122	22
Male	254	93
Grade		
G1	55	NA
G2	180	NA
G3	123	NA
G4	13	NA
unknow	5	NA
Stage		
I	175	55
II	86	35
III	86	31
IV	5	3
unknow	24	1
T classification		
T1	185	NA
T2	94	NA
T3	81	NA
T4	13	NA
TX	1	NA
unknow	2	NA
M classification		
M0	272	NA
M1	4	NA
MX	100	NA
N classification		
N0	257	NA
N1	4	NA
N2	114	NA
unknow	1	NA
Survival status(OS)		
Death	132	23
Survival	244	92

NA: Not available.

In addition, we downloaded the LIRI-JP data from the International Cancer Genomics Consortium (ICGC) database(https://dcc.icgc.org/).

### Differential expression analysis and identification of prognostic-related CRGs

We used Wilcoxon rank-sum test for differential analysis to identify differential expression levels of cuproptosis-related genes (CRGs) between HCC samples and non-tumor samples. Kaplan-Meier (KM)analysis and univariate Cox regression were then used to further determine the CRGs associated with prognosis.

### Consensus clustering analysis of CRGs GSVA and ssGSEA

Consensus clustering analysis, cumulative distribution function (CDF), and consensus matrix were performed to determine the optimal number of types. The correlation between types, overall survival (OS) status, and risk score was explored by the “GGalluvial” R package. Gene Set variation analysis (GSVA) analysis of pathway differences between different types. Then we assessed immune cell infiltration in different classifications using ssGSEA analysis.

### The intersection of genes and enrichment analysis

We integrated TCGA liver cancer data with GSE76427 data. We considered it as statistically significant when |log2(fold change) | > 0.585 and adjust P value< 0.05.Next, we used the “org.Hs.eg.db” and “enrichplot” packages to perform the Gene Ontology (GO) enrichment analyses to explore the relevant biological functions and structures, and the related pathways were obtained using the Kyoto Encyclopedia of Genes and Genomes (KEGG) enrichment analyses.

### Signature generation and validation

We obtained univariate significant genes by univariate Cox regression analysis. Next, LASSO Cox regression analysis was performed on univariate significant genes to minimize the risk of overfitting between signatures (28, 29). Multivariate Cox regression further screened out the four best genes for risk model construction and calculated their correlation coefficients. Then we calculated the risk score for each patient using the following formula: Riskscore $=\sum_{i=1}^{n}\exp\left(\text{Xi}\right)\times coef\;\left(\text{Xi}\right)$ ,here “exp(Xi)”, “coef(Xi),” and “n” represented the expression level, the coefficient, and the four genes, respectively.

Based on the median risk score of the training group, all HCC patients were divided into a high-risk group and a low-risk group. Log-rank test was used to analyze the different OS between high-risk and low-risk groups. The sensitivity and specificity of the signature were assessed by time-dependent receiver operating characteristics (ROC) analysis. Next, we constructed the programs using risk score, age, sex, and clinical stage. In addition, we plotted the calibration curves for years 1,3, and 5 to verify the accuracy of the nomogram. Furthermore, we analyzed the prognostic differences between subgroups stratified by age, gender, and clinical stage.

### Assessing the tumor microenvironment, tumor mutation burden correlation, and immune checkpoints

We used the ESTIMATE algorithm to assess the immune score, stromal score, and ESTIMATE score in the tumor microenvironment (TME) (30, 31). The algorithm was able to estimate the levels of stromal cells and immune cells in malignant tumor tissues using gene expression signatures. The ESTIMATE algorithm is implemented using the R package (estimate, https://sourceforge.net/projects/estimateproject/). The proportion of the corresponding component in the TME is indicated by the score. The TMB scores for each HCC patient in the TCGA cohort were assessed using somatic mutation analysis. We constructed correlation scatter plots and boxplots based on Pearson correlation analysis to search for the effect of risk score on TMB. Waterfall plots regarding high- and low-risk groups were generated by R package “maftools”. We identified m6A genes and potential immune checkpoints based on previously published literature (32–35).

### Evaluation of drug sensitivity and efficacy of immunotherapy

We used the R package “pRRophetic” to measure the 50% maximum inhibitory concentration (IC50) of different groups of samples by ridge regression to predict chemotherapeutic sensitivity (36). Wilcoxon sign-rank test was used to compare the IC50 of different groups. Next, we used the Tumor Immune Dysfunction and Exclusion (TIDE) Tool to predict immunotherapy responsiveness(http://tide.dfci.harvard.edu/).

### Tissue specimens and immunohistochemical staining

We collected tissue samples from 16 HCC patients at the Second Affiliated Hospital of Nanchang University. This study was reviewed by the Medical Ethics Committee of the Second Affiliated Hospital of Nanchang University. Patients included in this experiment were informed and written consent was obtained, and this study met the criteria set by the Declaration of Helsinki. Tissue specimens were fixed with 4% paraformaldehyde and then embedded in paraffin. Slice the tissue into 5 μm slices using a slicer. This was followed by dewaxing with xylene and water incorporation with ethanol solutions of varying concentrations for antigen repair. They were then sealed with 10% goat serum. Then we used anti-TAF6(1:100) to incubate overnight at 4°C. Following three washes, slides were incubated with secondary antibody for 30 mins at 25°C. After incubation, DAB was used to stain for 10 min, and hematoxylin was re-stained for 2 min.

### Cell culture and transfection

The human HCC cell line (HCCLM3) was purchased from the Shanghai Institute of Cell Research, Chinese Academy of Sciences, and the cells were validated by the cell bank of short tandem repeats. The HCCLM3 cell lines were cultured in DMEM culture media with penicillin G (100 μg/mL), streptomycin (100μg/mL), and 10% fetal bovine serum (FBS; Gibco; USA), and the cells were grown at 37°C with 5% CO_2_. The logarithmic growth cells were taken for the experiment. We used Lipofectamine 3000 Transfection Reagent (Invitrogen, Waltham, Massachusetts, USA) and interfering fragment siRNA and negative control si-NC (Hanheng Biotechnology Co. Ltd. Shanghai, China) to make transfection in HCCLM3 cells based on provided directions. Western blot (WB) and qRT-PCR were used to detect cell transfection efficiency. The sequences of siRNA are shown in **Table S1**.

### Quantitative real-time PCR and protein extraction and western blot

First, we used the Trizol method to extract total RNA from tissues and cells. Next, we reverse transcribed it into cDNA (TaKaRa, RR047A) and used it for real-time quantitative PCR(TAKARA, RR420A). Data analysis was performed using the 2^-ΔΔCt^ method. The primer sequences used are listed in **Table S1**. Total protein from HCCLM3 cells transfected with or without si-TAF6 was extracted, and western blotting was performed using the following primary antibodies:anti-TAF6 (1:500, Bioss, Beijing, China), anti-PD1/CD279 (1:2000, Proteintech, Wuhan, China) and anti-GAPDH (1:8000, Proteintech, Wuhan, China).

### Cell counting kit-8 assay and EdU assay

The proliferative capacity of HCCLM3 with/without TAF6 downregulation was observed by CCK-8 assay and EdU assay. Three group cells were seeded in 96-well plates at 3×10^3^ cells per well for the CCK-8 assay. Cell proliferation was detected using a CCK8 kit(Bioss, Beijing, China). 10μl CCK-8 reagent was added to each well at 0h, 24h, 48h, and 72h after transfection, and the cells were incubated at 37°C for 1.5h without light. The absorbance was measured at 450nm and we repeated each experiment at least three times. In addition, three group cells were seeded in 96-well plates at 1.5×10^4^ cells per well for EdU assay. According to the instructions of the YF^®^594 Click-iT EDU(UE, Shanghai, China) staining kit, the EDU was diluted to 50 μmol/L by the complete medium. Then we added 100 μL to each well and incubated for 2 h. Next, the medium was removed, and the cells were fixed in 4% paraformaldehyde and neutralized with 2mg/mL glycine solution, and washed twice with 3%BSA. 0.5%Triton X-100 was used as the osmotic enhancer, and the required Click-iT working solution was configured and incubated for 30min under dark conditions.1×Hoechst 33342 solutions were used for Nuclear redyeing. Finally, Images were taken with a fluorescence microscope and analyzed with Image J(version1.8).

### Wound healing assay and transwell migration assay

The migration ability of HCCLM3 with/without TAF6 downregulation was observed by wound healing assay and transwell migration assay. The cells were digested, centrifuged, resuspended, and counted. Then seed plate in a 6-well plate by 6×10^5^ per well. Then the cells were placed in incubators and incubated. When the monolayer was adherent to the wall, the scratch test was performed with a 200μL sterile pipette. Then the cells were washed with PBS 3 times, added to a serum-free medium, and placed in an incubator at 37°C. Finally, images were taken at 0h, 24 h, and 48h with a microscope and analyzed by ImageJ. In addition, HCCLM3 cells with/without TAF6 downregulation were seeded in transwell chambers at 2×10^4^ cells per group. Serum-free medium was used for the upper layer of the chamber, and a complete medium with 10% FBS was used for the lower layer of the chamber. Then we cultured HCCLM3 cells at 37°C with 5% carbon dioxide. After 24 hours of culture, wash, fix and stain. Finally, images were taken with a microscope and analyzed with ImageJ(version1.8).

### Statistical analysis

We performed a descriptive statistical analysis of HCC patients in TCGA and GEO. Continuous variables were described as the mean ± standard deviation, and categorical variables were described as frequency and proportions. The Kruskal-Wallis rank-sum test was applied to examine the differences in CRGs and DECRGs expression in the various classifications of the pathological stage and histological grade of HCC patients. The chi-square test was used to analyze clinicopathological features between the training and test set. The log-rank test was used to compare the OS and the median OS. The Wilcox test was used to assess the correlation between signature genes and immune checkpoint expression levels. All statistical analyses were performed using R version 4.1.3 and GraphPad Prism 8. P< 0.05 represented a statistical difference.

## Results

### Differential expression and genetic alterations of CRGs

**Figure 1:**
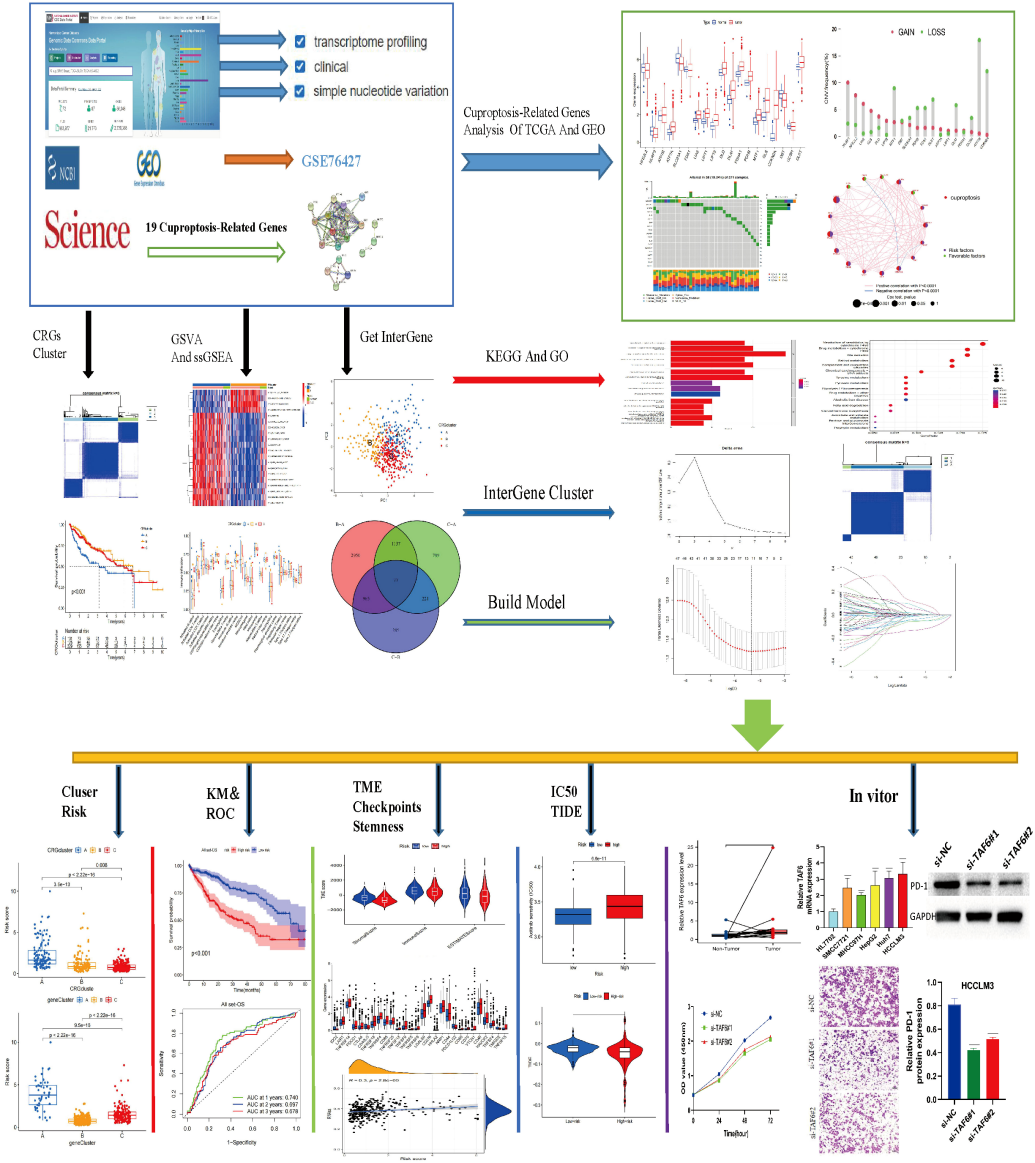
Flow chart of this study.

**Figure 2:**
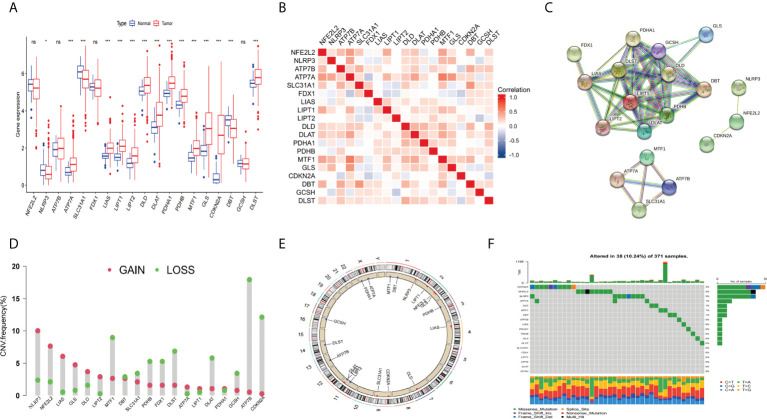
Expression and genetic alteration of CRGs in HCC. **(A)** the expression of 19 CRGs in HCC and normal tissues. **(B)** correlations between the expression of CRGs; **(C) **Protein-protein interaction (PPI) networks between CRGs; **(D–F)** the CNV and mutation frequency and classification of 19 CRGs in HCC. *p<0.05, wfi 2***p<0.001; ns, not statistically different.

The general process of this study is shown in **Figure 1**. First, we obtained 19 CRGs (NFE2L2, NLRP3, ATP7B, ATP7A, SLC31A1, FDX1, LIAS, LIPT1, LIPT2, DLD, DLAT, PDHA1, PDHB, MTF1, GLS, CDKN2A, DBT, GCSH, DLST) from previous studies (37). Next, we analyzed the differentially expressed genes of the CRGs between the tumors and the normal tissues of the HCC patients in the TCGA database. The results showed that APT7A, LIAS, LIPT1, LIPT2, DLD, DLAT, PDHA1, PDHB, MTF1, GLS, CDKN2A, and DLST showed significantly higher expression in tumor tissues than in normal tissues, and NLRP3, SLC31A1, and DBT had higher expression levels in normal tissues(**Figure 2A**). The correlation analysis of CRGs expression showed a strong positive relationship between MTF1 and ATP7A (**Figure 2B**). Next, we constructed the protein-protein interaction (PPI) networks(**Figure 2C**) by String (https://string-db.org/). We performed the copy number variation(CNV) frequency analysis of CRGs. The results showed that the increasing frequency of ATP7B and CDKN2A copies was significantly higher than the deletion frequency, and the deletion frequency of NLRP3 and LIAS copies was significantly higher than the deletion frequency (**Figures 2D, E** **)**. We also found that 38 somatic cells (10.24%) out of 371 liver cancer samples had mutations and CDKN2A (3%) showed a higher mutation frequency (**Figure 2F**).

**Figure 3:**
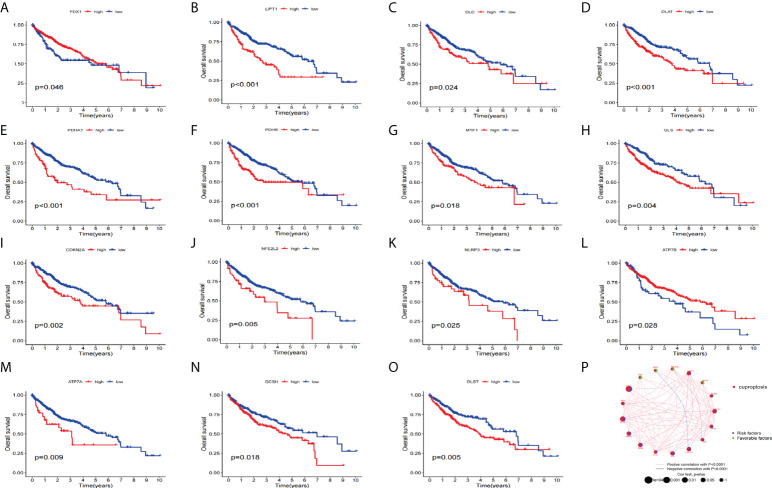
Prognosis significance of CRGs of HCC patients in TCGA and GEO in HCC. **(A–O) **K-M survival curve displays the OS of HCC patients. **(P)** Prognostic network of CRGs.

Next, we used KM analysis and COX analysis to assess the prognosis significance of CRGs of HCC patients in the TCGA and GSE76427 datasets collection files. Studies showed that 15 genes in the KM analysis were associated with OS prognosis (**Figures 3A–O**). The COX analysis showed those genes were associated with HCC patients (**Table 2**). Prognostic network maps of CRGs indicate LIAS, FDX1, SLC31A1, and ATP7B as protective factors in HCC, while NFE2L2, NLRP3, ATP7A, LIPT1, DLD, DLAT, PDHA1, PDHB, MTF1, GLS, CDKN2A, DBT, GCSH, and DLST are the risk factors (**Figure 3P**).

**Table 2:** UniCOX and KM analysis of CRGS.

id	HR	HR.95L	HR.95H	pvalue	km
LIPT1	2.041268484	1.400769396	2.974634537	0.000203862	6.66E-06
DLAT	1.318058945	1.107153366	1.569140677	0.001908279	0.000401478
GLS	1.199420121	1.043613805	1.37848754	0.010429257	0.003546961
ATP7A	1.409013614	1.05092514	1.889115873	0.021906859	0.008726055
PDHB	1.422772785	1.046290093	1.93472385	0.024543058	0.000414516
CDKN2A	1.151808608	1.016979706	1.304512826	0.026078951	0.002017522
NFE2L2	1.244314507	1.010434898	1.532328897	0.039620311	0.00505845
PDHA1	1.322017444	1.001537181	1.745047669	0.048745858	6.45E-05
MTF1	1.330351119	0.998711	1.772118361	0.051039522	0.018175397
GCSH	1.384991055	0.969040108	1.97948486	0.07387713	0.01826058
NLRP3	1.301558707	0.957318834	1.769582929	0.092635707	0.025387446
DLD	1.154216856	0.907978458	1.467233655	0.241409877	0.024130156
DLST	1.098050387	0.84537204	1.426253288	0.483289071	0.00517199
ATP7B	0.939395043	0.763851007	1.155281643	0.553616744	0.027650563
SLC31A1	0.969486093	0.782805565	1.200685491	0.776427617	0.065469526
DBT	1.015629815	0.791019231	1.304018766	0.903203134	0.16315505
FDX1	0.988440028	0.767063253	1.273706809	0.92838493	0.045997776
LIAS	0.992922625	0.694396004	1.419788324	0.96894854	0.079838689

### Division subtypes, GSVA, and ssGSEA analysis based on consensus cluster analysis

**Figure 4:**
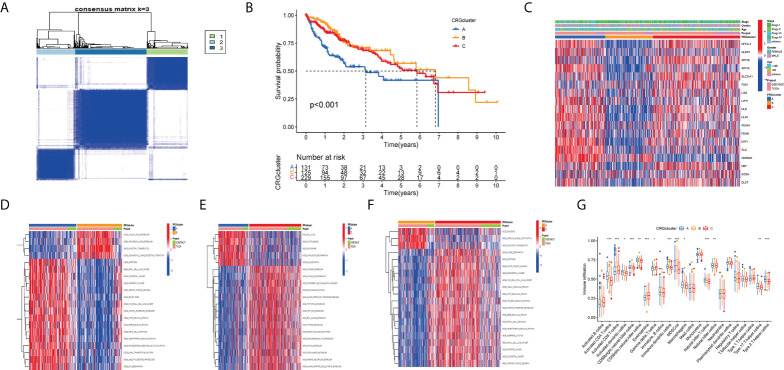
Clustering analyses of the signature. **(A, B) **Concordance matrix and K-M survival curve of the three clusters. **(C) **Complex heat maps show clinical correlations among the three clusters. **(D–F) **The GSVA heat map showed the differences in pathways in the three clusters. **(G) **The differential analyses between immune cells and the scale of fraction for cluster A and cluster B and cluster C. *p<0.05, **p<0.01, ***p<0.001.

The consistency matrix of the subtypes works best when K=3. Accordingly, we divided all HCC patients into three main subtypes (cluster A, cluster B, and cluster C) (**Figure 4A**). Next, we performed survival analysis on the three clusters and the KM curve showed significant differences in the prognosis between the different clusters, and the HCC patients with cluster B had the best prognosis (**Figure 4B**). A complex cluster-based heat map (**Figure 4C**) was constructed by combining the age, sex, and clinical stage of HCC patients in TCGA and GSE76427. Gene set variation analysis(GSVA) shows the differences between the top 20 most significant pathways in cluster A, cluster B, and cluster C (**Figures 4D–F**). The boxplot showed the difference in the proportions of different immune cells across the three clusters (**Figure 4G**).

### Acquisition of intersection genes and enrichment analysis

**Figure 5:**
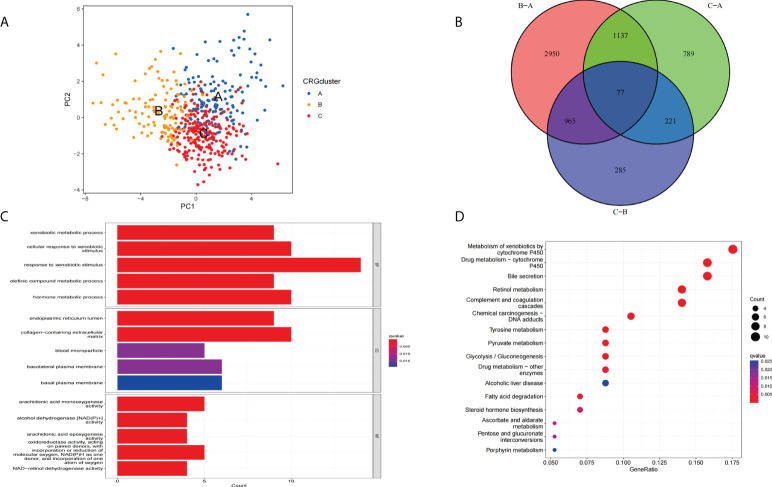
Functional enrichment analysis of DECRGs. **(A)** Principal component analysis of three clusters. **(B)** The Venn diagram shows the intersection of DECRGs **(C)** Analysis of BP, CC, and MF terms of GO enrichment demonstrated the possible function of the DECRGs. **(D)** Kyoto Encyclopedia of Genes and Genomes (KEGG) pathway enrichment analysis revealed the possible pathways.

The principal component analysis (PCA) results showed that we can segment cluster A, cluster B, and cluster C (**Figure 5A**) according to the expression level of CRGs. Next, we performed a differential analysis of the three clusters to obtain differentially expressed cuproptosis-related- genes (DECRGs) among different types. The Venn diagram shows the intersection of DECRGs (**Figure 5B**). Then, possible functions and pathways were identified using the GO and KEGG enrichment analysis. The GO analysis showed that the DECRGs were closely related to the endoplasmic reticulum lumen and basolateral plasma membrane in the Cellular Component (CC). Biological processes (BP) are mainly involved in the response to xenobiotic stimulus and regulation of body fluid levels. The Molecular Function (MF) can affect iron ion binding and monooxygenase activity (**Figure 5C**). The results of KEGG enrichment analysis showed that DECRGs are involved in chemical carcinogenesis−DNA adducts, alcoholic liver disease, xenobiotics by cytochrome P450, and drug metabolism − cytochrome P450, bile secretion, and retinol metabolism (**Figure 5D**).

### Consensus clustering analysis for partition subtype and prognostic model construction

First, we randomly divided 487 patients in a 1:1 ratio into the training set (n = 244) and the testing set (n = 243). Then we screened the 77 DECRGs by univariate Cox regression analysis to obtain the univariate significant genes (uniSigGenes) (**Supplementary S1**) and subtyped the uniSigGenes.

**Figure 6:**
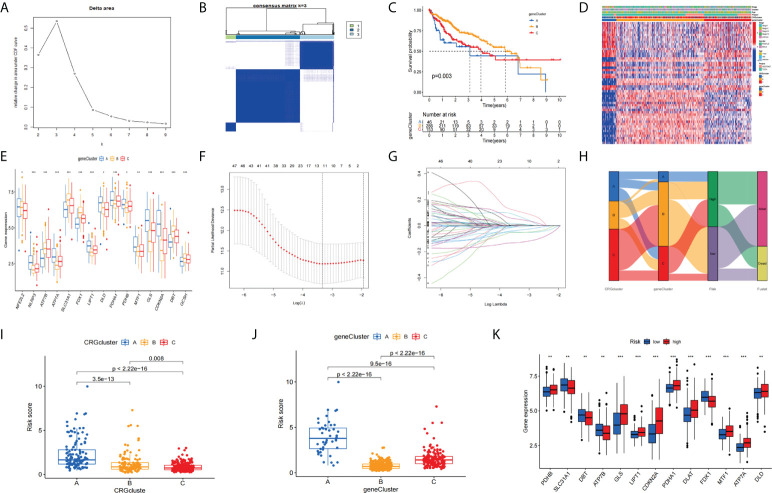
Clustering analyses of the signature. **(A) **The cumulative distribution function based on the sign indicated that the optimal number of subtypes was 3. **(B)** Concordance matrix of subtypes. **(C)** K-M survival curve of the three clusters. **(D)** A complex heat map illustrated the expression patterns. **(E)** Expression of CRGs between cluster A, cluster B, and cluster C **(H)** Ggalluvial shows the construction of the prognostic model. **(F, G)** LASSO regression analyses for screening LASSOSigGenes. Boxplots indicate the differences in risk scores in the CRG cluster **(I)** and the gene cluster **(J)**.The differential analysis of CRGs expression **(K)**. *p<0.05, **p<0.01, ***p<0.001.

Based on the k-value selected by the highest correlation coefficient, we classified all HCC patients into three major subtypes (cluster A, cluster B, and cluster C) (**Figures 6A, B** **)**. Patients in Cluster B had better OS compared to clusters A and clusters C (**Figure 6C**). A complex cluster-based heat map (**Figure 6D**) was constructed by combining the age, gender, and clinical stage of HCC patients in TCGA and GSE76427. Furthermore, we analyzed the differential expression of genes associated with CRGs between different clusters. The boxplot indicated that SLC31A1, FDX1, FDX1, and GCSH have the highest expression levels (**Figure 6E**) in cluster B. Next, we performed a LASSO regression analysis to reduce the overfitting of genes during signature generation and identified 11 significant genes (LASSOSigGenes) (**Figures 6F, G** **)**. Then, the multivariate COX regression analysis of the LASSOSigGenes was performed, which finally identified the best four genes (TAF6, SPP2, CFHR4, DNASE1L3).The data in the training set was used to build the prognostic model, and the signature formula is as follows: Risk score = expTAF6×0.257 + expSPP2× (-0.093) + expCFHR4× (-0.112) + expDNASE1L3× (-0.205). The 244 patients in the training set were divided into low and high-risk groups according to the median score calculated by the risk score formula. Then 243 patients were divided into low-risk and high-risk groups based on the median value of the risk score of the training group. Next, we combined the training set and the testing set files to get all set files. The Sankey diagram shows the construction of the prognostic model (**Figure 6H**). Boxplots indicate the differences in risk scores in the CRGs cluster (**Figure 6I**) and the gene cluster (**Figure 6J**). The differential analysis of CRGs expression in the collection file was performed and the boxplot results (**Figure 6K**).

### Validation of the prognostic value of the signature

**Figure 7:**
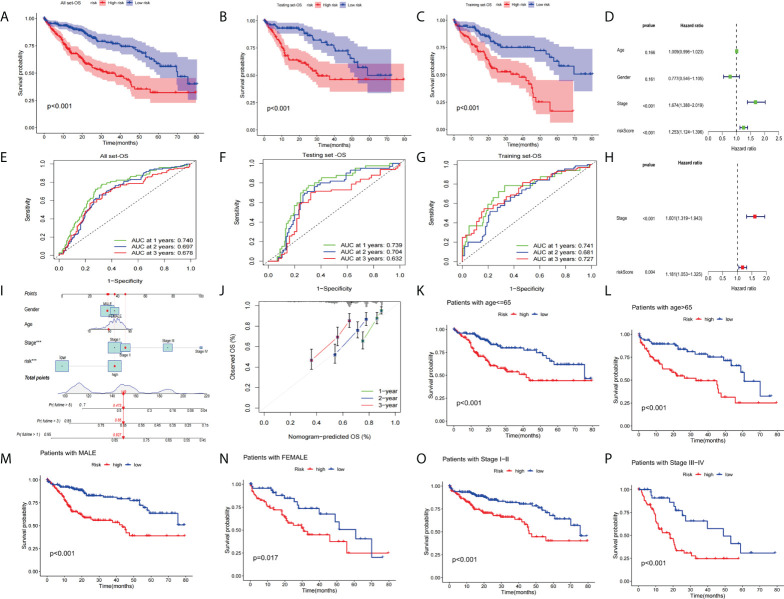
Validation of the prognostic value of the signatures. **(A–C)**The KM curve of all sets, testing set, and training set. **(D)** Univariate Cox regression analysis and multivariate Cox regression **(H)** analysis risk score and clinical stage as independent predictors of OS. **(E–G)** ROCs for 1-year, 2-year, and 3-year OS prediction. **(I)** The nomogram of the risk score and clinical parameters (age, gender, and stage) of all sets. **(J)** The calibration curves displayed the accuracy of the nomogram in the 1st, 2nd, and 3rd years. **(K, L)** Comparison of the OS between the high-risk and low-risk groups of patients who are<= 65 or > 65 years, male **(M)** or female **(N)** with a stage of stage I-II **(O)** or stage III-IV **(P)**.

We performed prognostic analysis on all set, training set, and testing set data. The KM curve indicates that the low-risk group showed a better OS probability (**Figures 7A–C**) compared with the high-risk group. In addition, we plotted 1-year, 2-year, and 3-year ROC curves to assess the sensitivity and specificity of the signature (all AUC > 0.600, **Figures 7E–G**). Univariate and multivariate COX regression analysis was performed in all sets. Univariate Cox regression analysis indicated that the risk score and clinical stage were significantly associated with OS (**Figure 7D**). Multivariate Cox regression analysis also identified risk score and clinical stage as independent predictors of OS (**Figure 7H**). Next, we constructed OS-related nomograms to test the proportional hazards hypothesis (**Figure 7I**) in the multivariate Cox model. The subsequent calibration curve further validated the accuracy of the nomogram (**Figure 7J**). Finally, we performed a clinically stratified analysis of clinical factors(age, gender, and tumor stage) to understand the applicability of the signature. We observed that for HCC patients aged<= 65 or > 65 years, the survival rate was significantly higher in the low-risk group than in the high-risk group (**Figures 7K, L**, all p< 0.001). Furthermore, for male or female HCC patients, the low-risk group was significantly higher than the high-risk group (**Figures 7M, N**, all p< 0.05). Similarly, for patients with tumor stage I-II (p< 0.001) or III-IV (p<0.001), survival in the low-risk group was significantly higher than in the high-risk group (**Figures 7P, O**).

**Figure 8:**
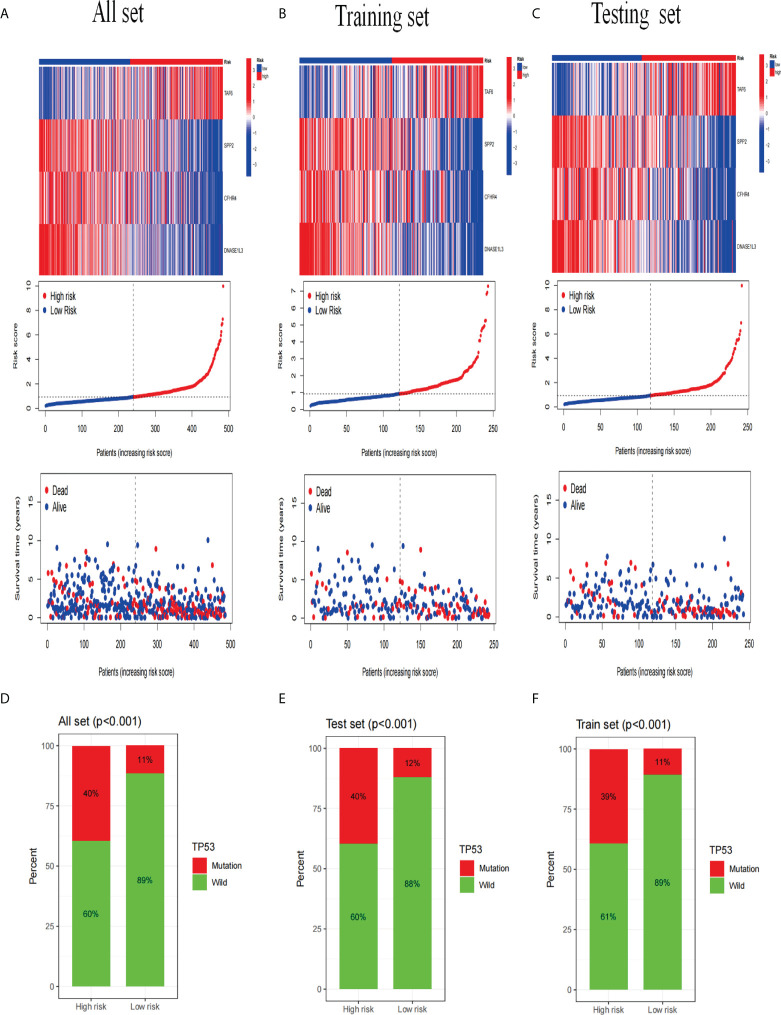
Analysis of gene expression and mutation correlation. **(A–C)** The risk curve consists of genes expression heat map, risk score curves, and survival status point plot. **(D–F)** Comparison of the proportion of the TP53 mutation status in the high- and low-risk groups in the training, testing, and all sets.

Next, we analyzed the expression differences of the four genes constructing the signature in the all set (**Figure 8A**), training set (**Figure 8B**), and testing set (**Figure 8C**). By mapping the heat map, we found that TAF6 was upregulated but SPP2, CFHR4, and DNASE1L3 had higher expression in the low-risk group. In addition, we also analyzed the mutation situation of TP53 with a higher mutation frequency in the three groups. We found that TP53 had a higher proportion of mutations in the high-risk group for the all-set (**Figure 8D**), training-set (**Figure 8E**), and testing-set (**Figure 8F**).

### Association of signature with immune cell infiltration and mutational load and immune checkpoints

**Figure 9:**
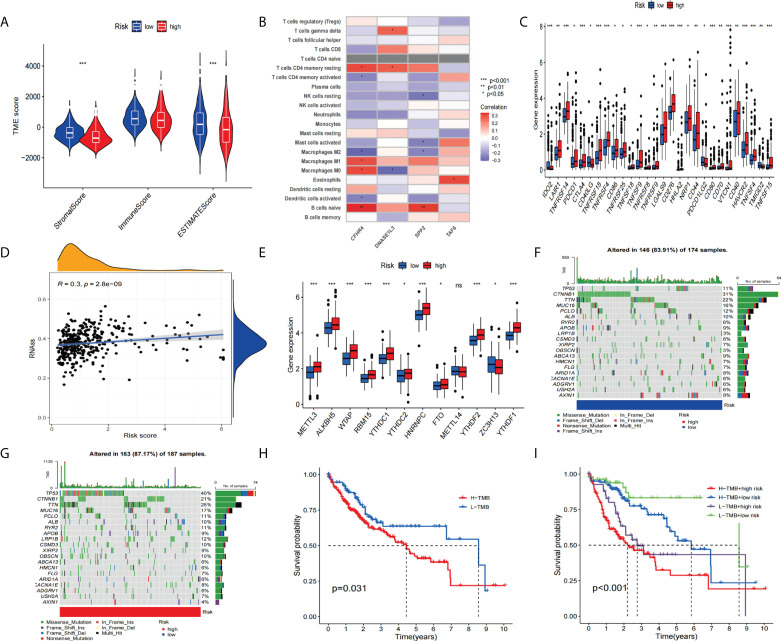
To assess the tumor microenvironment, immune checkpoint genes, and tumor mutation burden (TMB) in different groups. **(A)** Comparison of ESTIMATE scores, stromal scores, and immune scores between the high-risk and low-risk groups. **(B)** Correlation between the model-constructed genes and immune cells. **(C)** Differential expression of immune checkpoint genes between the high-risk group and the low-risk groups. **(D)** Correlation between the stem cell content and the risk score. **(E)** Differential expression of m6A-related genes **(F, G)** and the frequency of mutations in the high-risk and low-risk groups. **(H, I) **The KM curve of the tumor mutation burden versus the OS. *p<0.05, **p<0.01, ***p<0.001; ns, not statistically different.

Immunotherapy had revolutionized cancer treatment, enabling longer survival for cancer patients (38). However, immune cell infiltration and immune checkpoints are important factors in immunotherapy (39, 40). Therefore, we performed a multifaceted analysis of the relationship between signatures and immunity. The results showed that there was no difference between the two groups, and the stromal score and estimated score of the risk group were significantly higher than the high-risk group (**Figure 9A**). Next, we analyzed the correlation of the four genes with immune cells. The correlation heatmap (**Figure 9B**) shows the immune correlation results. Next, we analyzed the expression of the checkpoint genes between the high-risk and low-risk groups. The boxplot results showed that the expression of all checkpoint genes was statistically significant (**Figure 9C**), especially IDO2, TNFRSF14, CTLA4, TNFRSF18, TNFRSF4, LGALS9, CD276, HHLA2, CD80, VTCN1, HAVCR2, TNFSF4, and TNFSF15 (p<0.001). Studies have shown that the recurrence, metastasis, and chemotherapy resistance of liver cancer are associated with the presence of cancer stem cells (41).

So we performed a stem-cell correlation analysis. The correlation scatters plot (**Figure 9D**) indicates a higher risk score and a higher stem cell content. In addition, we analyzed the differences in m6A-related gene expression between the high-risk and low-risk groups. The results(**Figure 9E**) showed that METTL3, ALK8H5, WTAP, RBM15, YTHDC1, YTHDC2, HNRNPC, FTO, YTHDF2, and YTHDF1 showed higher expression in the high-risk group compared to the low-scoring risk group. Next, we analyzed the differences in mutation frequency in the high-risk groups and low-risk groups. The waterfall map shows (**Figures 9F, G** **)** that the mutation frequency of TP53 is much higher in the high-risk group than in the low-risk group. Furthermore, the KM survival curves showed a combination of higher TMB values and higher risk scores associated with worse OS (**Figures 9H, I** **)**.

### Signature predict the efficacy of response to chemotherapy and immunotherapy

**Figure 10:**
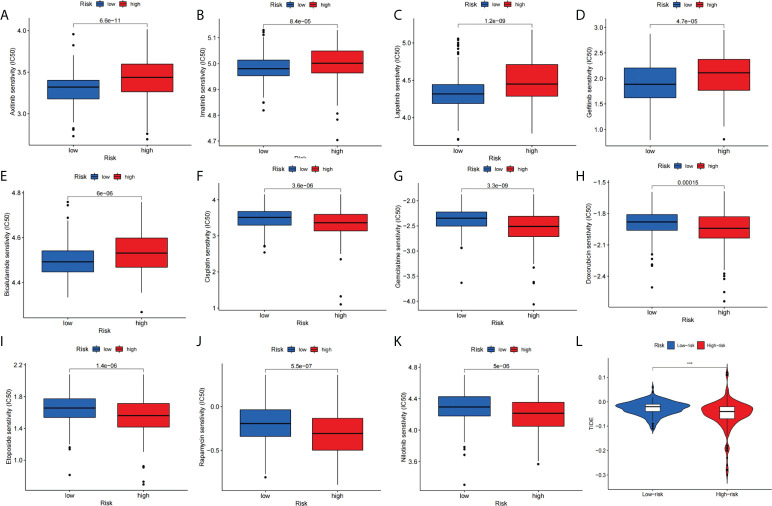
Signature predicts chemotherapy and immunotherapy response. **(A–E)** The signature showed high-risk scores were associated with a lower IC50 for chemotherapeutics such as **(A)** Axitinib, **(B)** Imatinib, **(C)** Lapatinib, **(D)** Gefitinib, and **(E)** Bicalutamide, whereas they were related to a higher IC50 for **(F)** Cisplatin, **(G)** Gemcitabine, **(H)** Doxorubicin, **(I)** Etoposide, **(J)** Rapamycin and **(K)** Nilotinib treatment. **(L)** Differences in TIDE score between high- and low-risk groups.

We compared the relationship between high-risk and low-risk populations and the efficacy of the drug to assess the predictive effect of this signature on HCC drug therapy. Our study showed that the low-risk group was significantly associated with higher IC50 with chemotherapy drugs such as Axitinib, Imatinib, Lapatinib, Gefitinib, and Bicalutamide (**Figures 10A–E**).In contrast, the high-risk group was more sensitive to Cisplatin, Gemcitabine, Doxorubicin, Etoposide, Rapamycin, and Nilotinib treatment (**Figures 10F–K**). Furthermore, we assessed differences in immunotherapy between high-risk and low-risk patients by TIDE score. The results showed that the TIDE score is significantly lower (**Figure 10L**) in high-risk patients compared with low-risk patients. Next, we used TIMER2.0 (http://timer.cistrome.org/) to perform the correlation analysis of the four genes constituting the signature with PDC1, LMTK3, and CTLA4 expression (42). After adjusted by purity, the results (**Supplementary Figures 1A–C**) revealed that the expression level of CFHR4, DNASE1L3, and SPP2 were significantly negatively correlated with PDCD1, LMTK3, and CTLA4 in HCC, but TAF6 was positively correlated with PDCD1, LMTK3, and CTLA4 in HCC.

### Differential expression analysis and prognostic analysis of the signature genes

**Figure 11:**
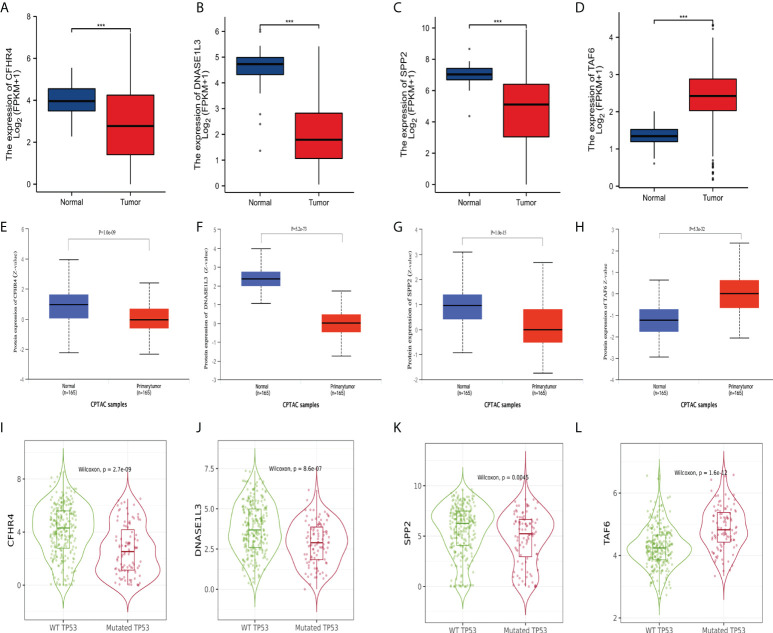
Differential expression of the signature genes. **(A–D)** Differential expression of CFHR4, DNASE1L3, SPP2, and TAF6 in adjacent non-tumor tissues and HCC tissues in the TCGA database. **(E–H)** Differential expression of CFHR4, DNASE1L3, SPP2, and TAF6 in adjacent non-tumor tissues and HCC tissues in the CPTAC dataset. **(I–L)** Differential expression of CFHR4, DNASE1L3, SPP2, and TAF6 of HCC patients in the wild TP53 group and the mutant TP53 groups. ***p<0.001.

To further explore the credibility of the signature. We performed the differential analysis of the expression of signature genes on data from TCGA and CPTAC (http://ualcan.path.uab.edu/analysis-prot.html). The analysis of TCGA data showed that (**Figures 11A–D**), CFHR4, DNASE1L3, and SPP2 were significantly higher in adjacent non-tumor tissues, while TAF6 was upregulated in HCC tissues. Then we analyzed the CPTAC dataset, and the results were consistent with previous studies (**Figures 11E–H**). Furthermore, we used TIMER2.0 to analyze the expression differences of the signature genes between the two groups of wild TP53 and mutated TP53 in HCC patients. Boxplots (**Figures 11I–L**) indicated that CFHR4, DNASE1L3, and SPP2 had higher expression levels in the wild TP53 group, while TAF6 showed higher levels in the mutated TP53 group.

Next, we analyzed the relationship of the label genes with the prognosis. We used Kaplan-Meier Plotter (http://kmplot.com/analysis/index.php?p=service) to analyze the association of the signature genes with overall survival (OS) in HCC patients. The KM (**Supplementary Figure 2A**) curve of OS showed that all four genes were significantly associated with the prognosis of HCC patients. We then performed a prognostic analysis of the liver cancer data (**Supplementary Figure 2B**) from the ICGC database. The results indicate that low expression of CFHR4 and DNASE1L3 is associated with poor prognosis in HCC patients, while high expression of TAF6 is associated with poor prognosis in patients. In addition, we analyzed the differential expression of the signature genes in the different histological grades, pathological stages, and T stages of HCC in the TCGA database. The expression levels of the four genes (CFHR4, DNASE1L3, SPP2, and TAF6) varied in the different histological grades, pathological stages, and T stages of HCC, except for DNASE1L3, which did not show any statistical differences between the T stages (**Supplementary Figure 3**).

### Validation of signature genes expression levels

**Figure 12:**
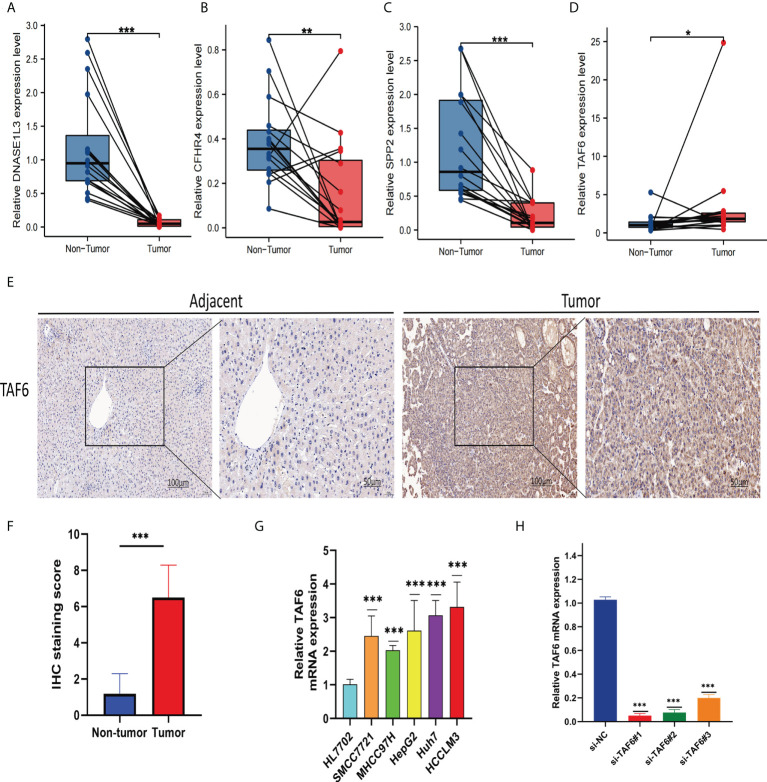
Verify the mRNA expression levels of the four signature genes in the tissues. The qRT-PCR results showed thatDNASE1L3 **(A)**, CFHR4 **(B)**, and SPP2 **(C)** were expressed higher in non-tumor tissues (Non-Tumor) than in tumor tissues (Tumor), TAF6 **(D)** was expressed higher in tumor tissues (Tumor)than in non-tumor tissues (Non-Tumor). TAF6 representative IHC **(E, F)** stained images in HCC tissue and adjacent tissues(n = 16; magnification: left, 100×; right, 200×). Expression levels of TAF6 **(G)** in HCC cell lines. The bar graphs **(H)** indicates better silencing for si-TAF6#1 and si-TAF6#2. *p< 0.05, **p< 0.01, and ***p< 0.001.

We collected tissue samples from 16 HCC patients for the analysis of signature gene expression levels at the Second Affiliated Hospital of Nanchang University. The real-time quantitative PCR results showed that CFHR4, SPP2, and DNASE1L3 were expressed higher in non-tumor tissues (Non-Tumor) (**Figures 12A–C**) than in tumor tissues (Tumor), and TAF6 was expressed higher in tumor tissues (**Figure 12D**) than in non-tumor tissues. Due to the importance of TAF6 in the four signature genes, we further evaluated it. IHC results showed that TAF6 was highly expressed in liver cancer (**Figures 12E, F**). These results further verified the correctness of the above bioinformatics research. In addition, we assessed the expression level of TAF6 in the cell lines in liver cancer cell lines. including HepG2,SMCC7721,MHCC97H,Huh-7 and HCCLM3.As shown in (**Figure 12G**), compared with HL7702 (normal liver cells), TAF6 was expressed at relatively higher levels in liver cancer cell lines. Next, we used the HCCLM3 with the highest TAF6 mRNA expression level for subsequent trials. The real-time quantitative PCR was used to test the knockdown efficiency. The bar graphs (**Figure 12H**) indicate better silencing for si-TAF6#1 and si-TAF6#2.

### TAF6 expression was associated with poor prognosis in HCC

**Figure 13:**
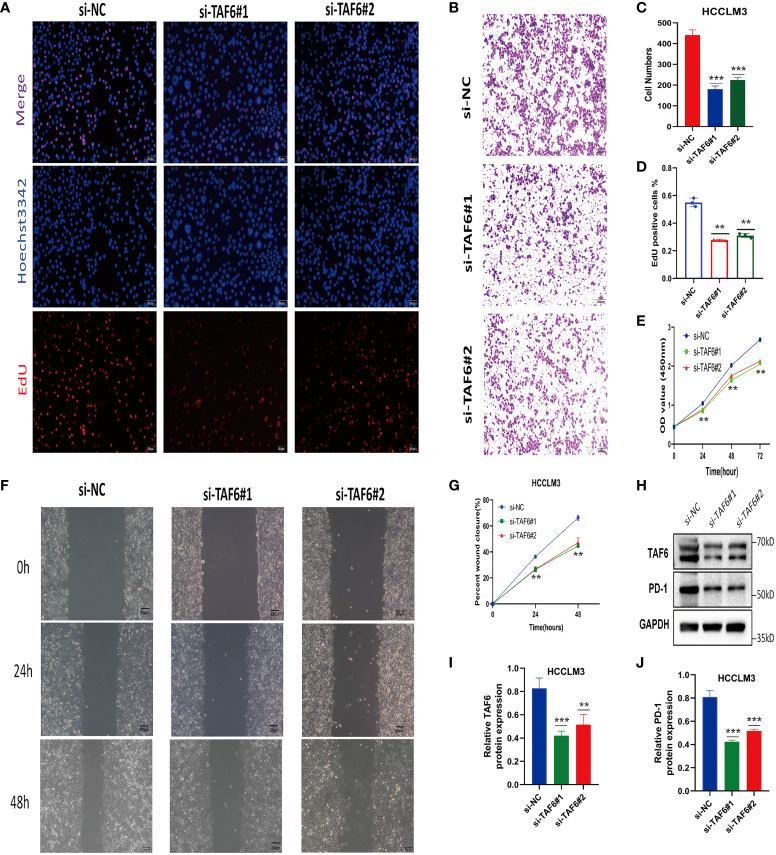
Adverse effects of TAF6 on HCC *in vitro*. **(A, D)** Compared with the control group, the proliferation rate of HCCLM3 cells was significantly inhibited after TAF6 silencing by EdU staining. **(B, E)** Transwell experiments showed that the migratory ability of HCCLM3 was inhibited after TAF6 silencing. **(C)** After TAF6 silencing, the cell viability of HCCLM3 was significantly inhibited by the CCK- 8 assay. **(F, G)** The wound healing array showed that LTAF6-downregulated HCCLM3 cells exhibited significantly delayed wound healing compared with controls. **(H–J)** Effects of with or without inhibition of TAF6 expression on PD-1 protein expression levels by western blotting. Scale bar: EdU,50μm; Transwell experiments and Wound healing array,200μm. **p<0.01, ***p<0.001.

The better knockdown si-TAF6#1 and si-TAF6#2 were used for subsequent trials. To assess the effect of TAF6 on proliferation in HCC, we used CCK-8 and EdU staining assays in HCCLM3 with/without TAF6 knockdown. After interfering with TAF6 expression in HCCLM3 cells, the cell proliferation rate in thesi-TAF6#1 and si-TAF6#2 groups was significantly lower than that in the si-NC group (**Figures 13A, D, E** **)**. The effect of inhibiting TAF6 expression on HCC cell migration was further analyzed. Transwell assays and wound-healing experiments together showed that the migratory ability of HCCLM3 cells was significantly reduced upon inhibition of TAF6 expression (**Figures 13B, C, F, G** **)**. Furthermore, we explored the relationship between the immune checkpoint PD-1 and TAF6 in HCC cells. Interestingly, we found that inhibiting the expression of TAF6 reduced the protein expression of PD-1 by western blotting (**Figures 13H–J**). In summary, *in vitro*, experimental data suggest that high expression of TAF6 is closely related to better immunotherapy results and poor prognosis in patients with HCC.

## Discussion

The incidence of liver cancer has been on the rise in recent years, and it is estimated to exceed 1 million cases by 2025 (43, 44). As the major histological type of liver cancer, hepatocellular carcinoma (HCC) accounts for the vast majority of liver cancer diagnoses and death (45). Although enhanced diagnostic techniques and treatments have enabled improved outcomes for early-stage HCC patients, the overall prognosis of HCC remains poor (46). Therefore, seeking a valid signature is significant for evaluating the prognosis and treatment of patients with HCC. Recent studies have shown that cuproptosis is a new form of programmed cell death, different from oxidative stress-related cell death, such as apoptosis, ferroptosis, and necroptosis (9). Studies have shown that copper death-related genes play important roles in clear-cell renal cell carcinoma (47), neuroinflammation (48), and chemotherapeutic drugs (49). In addition, numerous studies have shown the importance of pyroptosis (50), ferroptosis (51), and necroptosis (52) in the development and treatment of HCC. However, no study has analyzed the relationship between cuproptosis and prognosis prediction and targeted therapy in HCC patients.

In this study, we performed a clustering analysis of cuproptosis -related genes(CRGs) that yielded differentially expressed cuproptosis-related- genes (DECRGs). Then, the univariate regression analysis, LASSO Cox regression analysis, and multivariate regression analysis yielded four DECRGs. Next, we used four DECRGs to construct a novel prognostic risk signature and identify potential molecular subtypes to better predict the prognosis of HCC. Furthermore, we evaluated the performance of the signature through survival analysis, mutation correlation analysis, and independent prognostic analysis. In conclusion, our results indicate that the signature is better predictive. Our signature genes include TAF6, SPP2, CFHR4, and DNASE1L3, all of which are cuproptosis -related differential genes with a strong correlation with cuproptosis –related- genes (**Supplementary Figure 4**). Our signature genes have the potential to be a prognostic risk gene for HCC patients. Xiao et al. showed that DNASE1L3 inhibits HCC progression by inducing apoptosis and weakening glycolysis (53). Lu et al. found that SPP2 is involved in regulating aerobic glycolysis and affecting HCC tumorigenesis (54). Fan et al. have suggested that SPP2 can serve as a biomarker to predict the prognosis of patients with oral squamous cell carcinoma (55). The study of Wang et al. has demonstrated the great potential of TAF6 in the development of glioblastoma therapy (56). Furthermore, we analyzed the differential expression of signature genes in the TCGA database of genes and HCC data in the GSE76427 datasets and found significant differences in signature genes between adjacent non-tumor samples and HCC samples. Our results from qRT-PCR have also demonstrated the same results, with the GEPIA2 and ICGC data survival analysis results indicating those signature genes are significantly associated with prognosis, increasing the confidence of our signature genes as prognostic models. Based on the regression coefficients, TAF6 is considered the most important DECRG in risk factors and prognosis prediction. We analyzed the expression of TAF6 in normal hepatocytes and hepatoma cell lines, and the results of real-time quantitative PCR indicated that TAF6 expression was significantly higher in hepatoma cell lines than in normal hepatocytes. In addition, we performed *in vitro* experiments and found that interference with TAF6 expression significantly inhibited HCC proliferation and migration. Interestingly, we found for the first time that TAF6 inhibition causes downregulation of CD279 (PD-1) protein expression, which may provide new insights into immunotherapy

Our GO enrichment analysis indicates that the presence of DECRGs mainly in the basolateral plasma membrane and in the ER lumen may be associated with ER stress in regulating tumor growth and antitumor immunity (57). It may also affect immune responses by affecting iron ion binding (58). Accordingly, we performed the immune correlation analysis, and the results showed that there was no difference in the immune scores for the data from the high-risk and low-risk groups, and the significant differences in the stromal and estimated scores. This suggests that our DECRGs have the potential to predict the composition of the TME. The risk score was positively correlated with T cells CD4 memory activated and NK cells resting, and negatively correlated with T cells CD4 memory resting and B cells naive (59, 60) (**Supplementary Figure 5**). These results suggest that the high-risk group may have better immune cell therapy effects. Furthermore, the results of KEGG enrichment analysis indicated that DECRGs are involved in chemically oncogenic DNA adducts, alcoholic liver disease, and drug metabolism cytochrome P450. This result suggests that our signature may be used for the treatment of HCC and for developing chemotherapy drugs (61–63). Given the therapeutic importance of drug therapy for HCC, we evaluated the predictive effect of the signature on drug therapy for HCC. Our study showed that Cisplatin (64), Gemcitabine (65), Doxorubicin (66), Etoposide (67), Rapamycin (68), and Nilotinib (69)have higher drug sensitivity in the high-risk group, consistent with previous studies suggesting that our signature has the potential to predict drug efficacy. TMB has been proven to be an important and independent biomarker that plays an important role in the prognosis and treatment of HCC (70, 71). Our study showed that HCC patients in the high-score-risk group had a higher frequency of TP53 mutations and a worse prognosis, which is consistent with the study by Tang et al (72).In recent years, the use of immunotherapy in the treatment of HCC has attracted increasing attention (73), and checkpoint inhibitor immunotherapy plays an important role in HCC patients (74). We analyzed the expression levels of checkpoint genes and the TIDE scores in the high-risk and low-risk groups. The results showed that the checkpoint gene expression levels were generally higher in the high-risk group than in the low-risk group, while the high-risk patients had significantly lower TIDE scores. This suggests that our signature may be used to evaluate the expression of immune checkpoint genes and the effect of immunotherapy. Furthermore, we used TIMER2.0 to analyze the correlation analysis of the signature genes with the immune checkpoint genes PDCD1, LMTK3, and CTLA4. The results showed a significant correlation of the signature genes with both PDCD1, LMTK3, and CTLA4.In conclusion, our signature has a good predictive prognostic power and facilitates the selection of patients suitable for immunotherapy.

However, our study also has limitations. First, most of our data are derived from the TCGA, GEO, and ICGC databases, and we find a lack of comprehensive validation from the external datasets. Furthermore, we performed partial expression volume validation at the tissue level and cell level but the sample number was small. In the future, we will continue to collect samples to evaluate this signature with immunotherapy and to verify whether there are differences in immunotherapy benefits between high-risk and low-risk populations.

In conclusion, we constructed a new prognostic CRGs signature to better predict the prognosis in HCC. This signature will help clinicians to evaluate the overall patient prognosis and provide new ideas for developing treatment strategies.

## Data availability statement

The original contributions presented in the study are included in the article/**Supplementary Material**. Further inquiries can be directed to the corresponding authors.

## Ethics statement

This study was reviewed and approved by Medical Ethics Committee of the Second Affiliated Hospital of Nanchang University. The patients/participants provided their written informed consent to participate in this study.

## Author contributions

XP conducted the formal analysis and wrote the original draft. YL and RY performed the project administration. SL and JZ conducted the experiments. CL and XW participated in software analysis. XP, JZ, and ZL conducted data curation. XP, JZ, and RY contributed to writing, reviewing, and editing the article. RY provided funding acquisition. All authors contributed to the article and approved the submitted version.

## Funding

This study was supported by the National Natural Science Foundation of China (Nos.81960436, 81560396 and 82260460) the Project of the Jiangxi Provincial Department of Science and Technology (Nos. 20202BBGL73037 and 20192BCB23023), Double Thousand Talents Project of Jiangxi Province (No.jxsq2019201100).

## Conflict of interest

The authors declare that the research was conducted in the absence of any commercial or financial relationships that could be construed as a potential conflict of interest.

## Publisher’s note

All claims expressed in this article are solely those of the authors and do not necessarily represent those of their affiliated organizations, or those of the publisher, the editors and the reviewers. Any product that may be evaluated in this article, or claim that may be made by its manufacturer, is not guaranteed or endorsed by the publisher.
