# Supplementary figures and images for: Signature construction and molecular subtype identification based on cuproptosis-related genes to predict the prognosis and immune activity of patients with hepatocellular carcinoma
Source: Front Immunol. 2022 Sep 28;13:990790. doi: 10.3389/fimmu.2022.990790 (PMC9555242; doi:10.3389/fimmu.2022.990790)

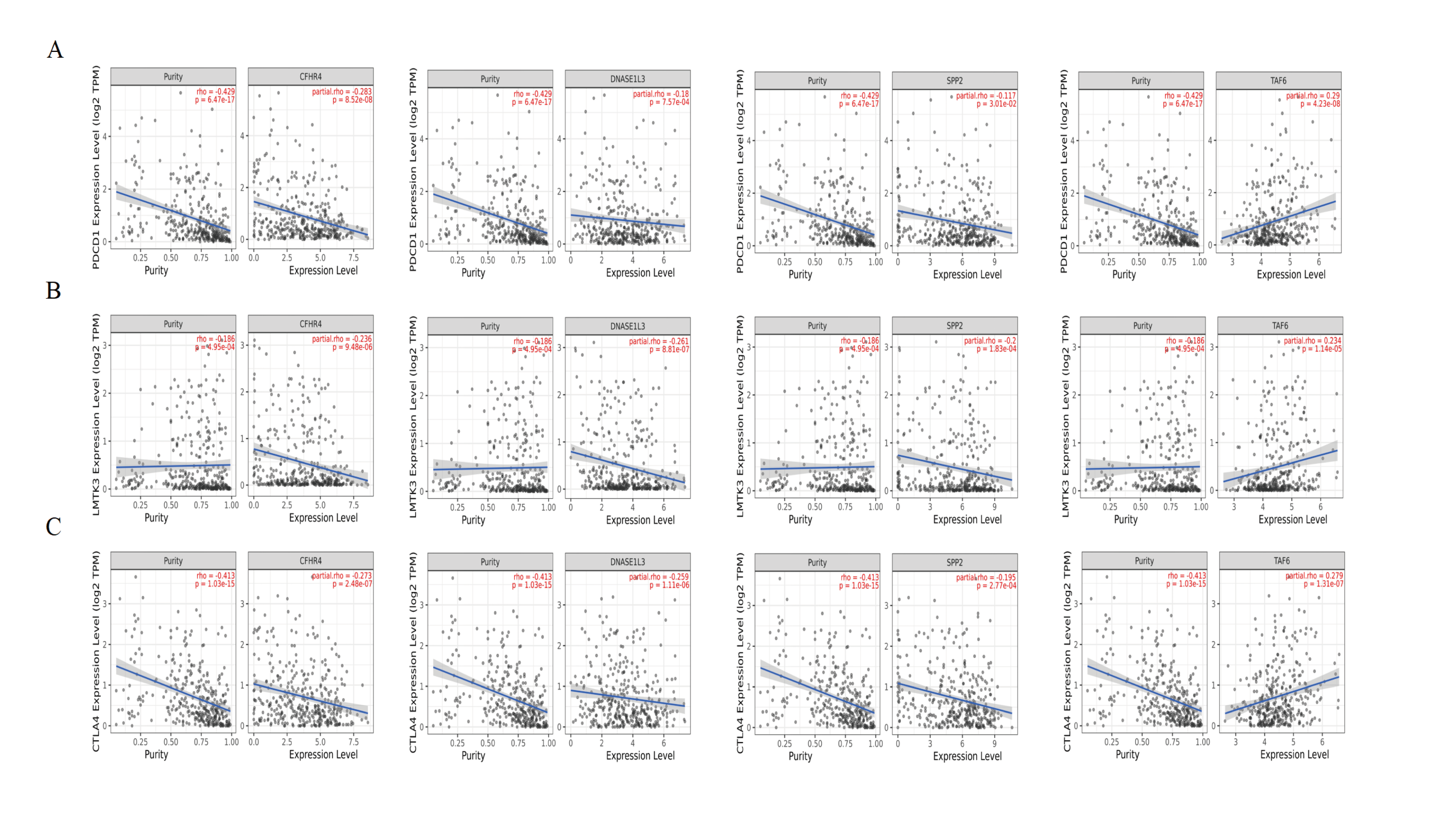

Supplement: Supplementary file 1 [file Image_1.tif]

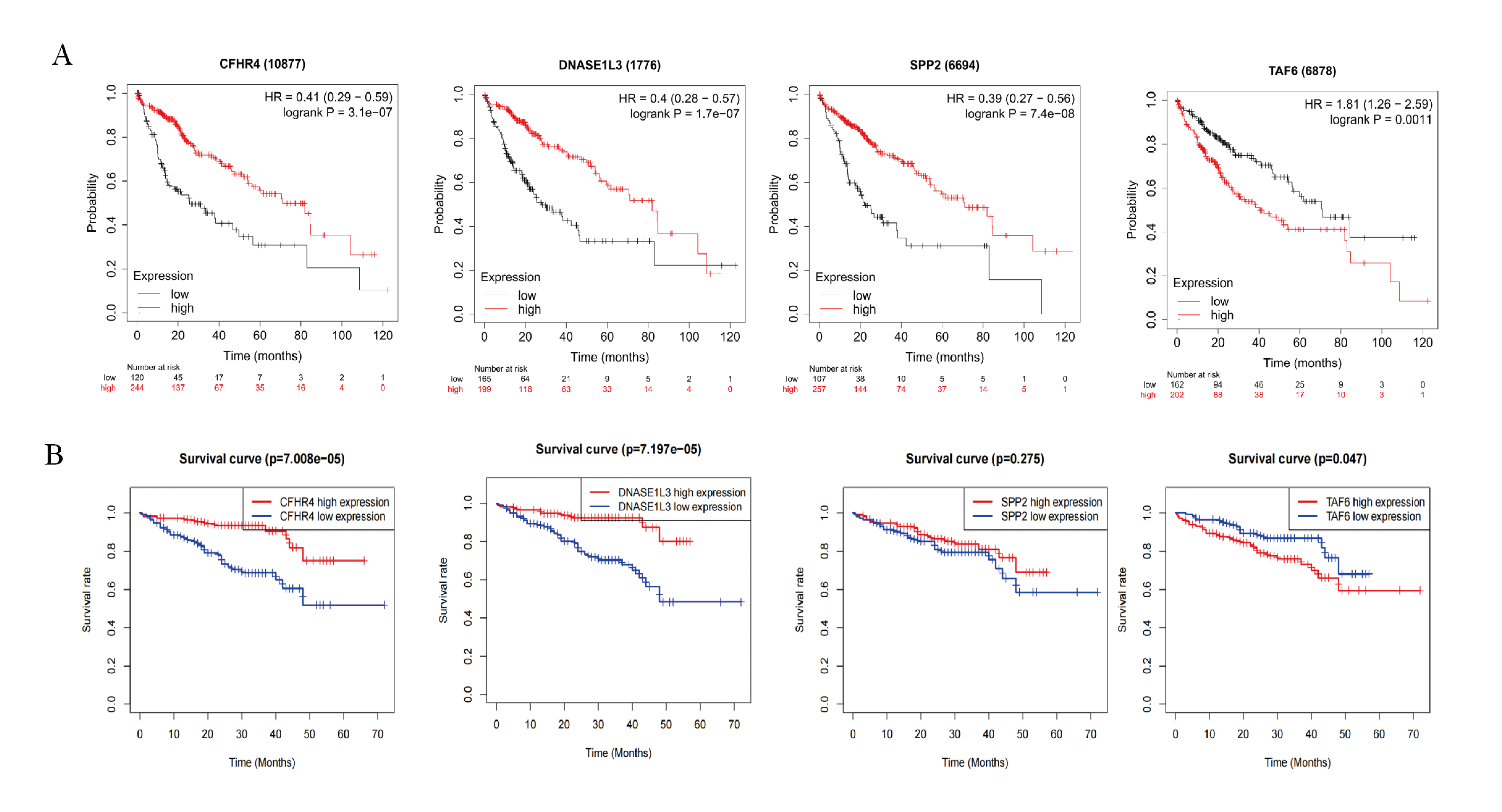

Supplement: Supplementary file 2 [file Image_2.tif]

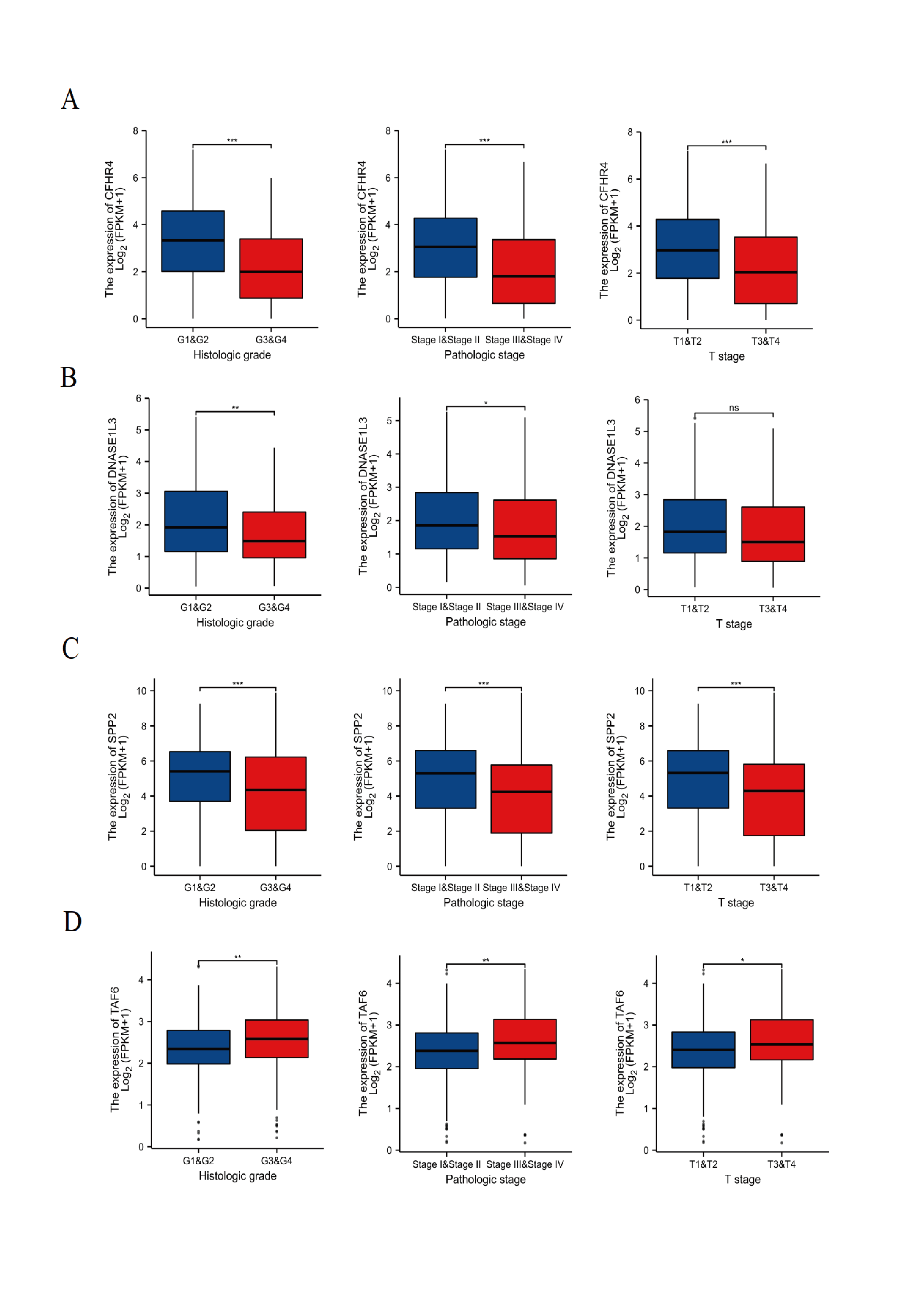

Supplement: Supplementary file 3 [file Image_3.tif]

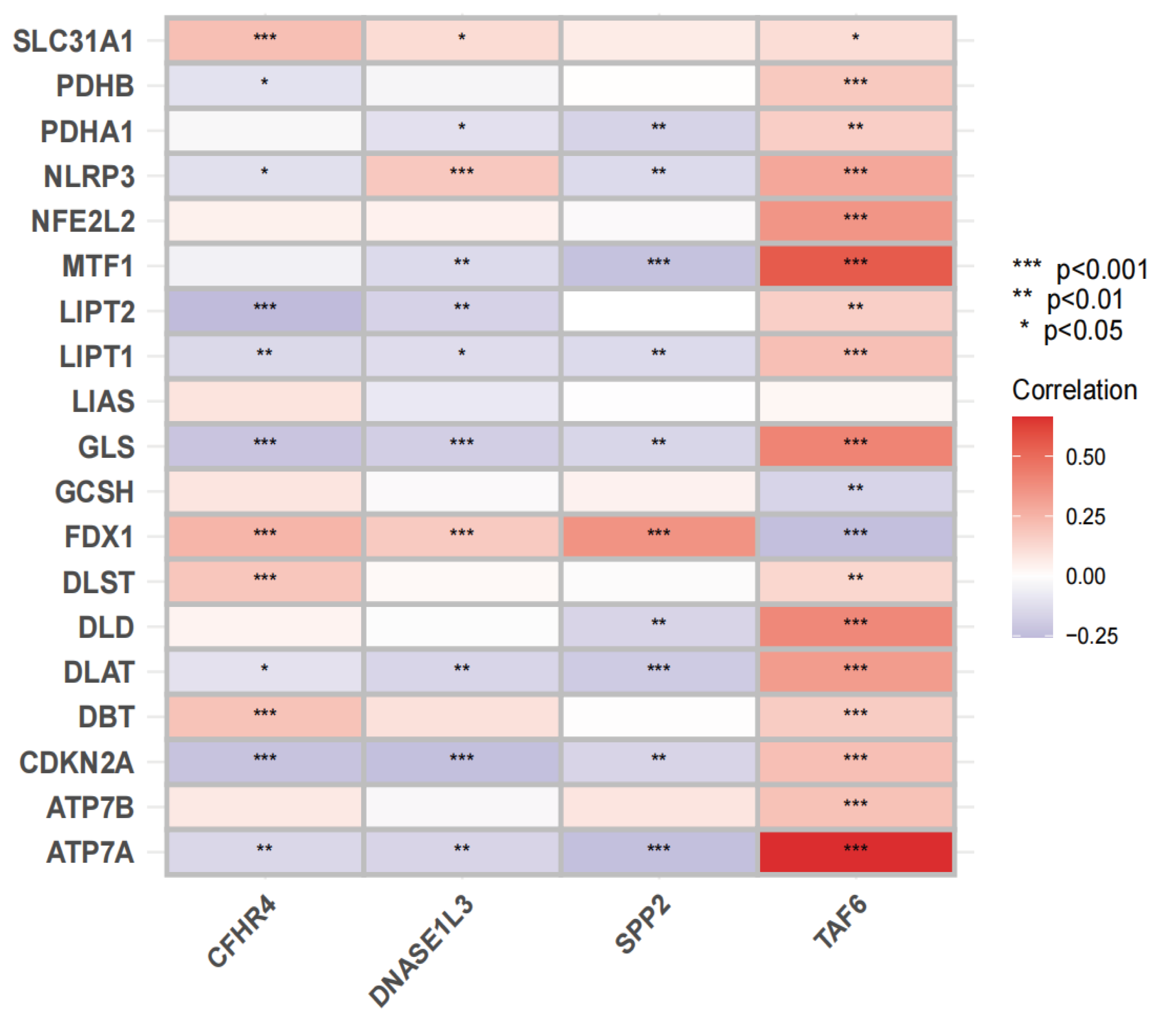

Supplement: Supplementary file 4 [file Image_4.tif]

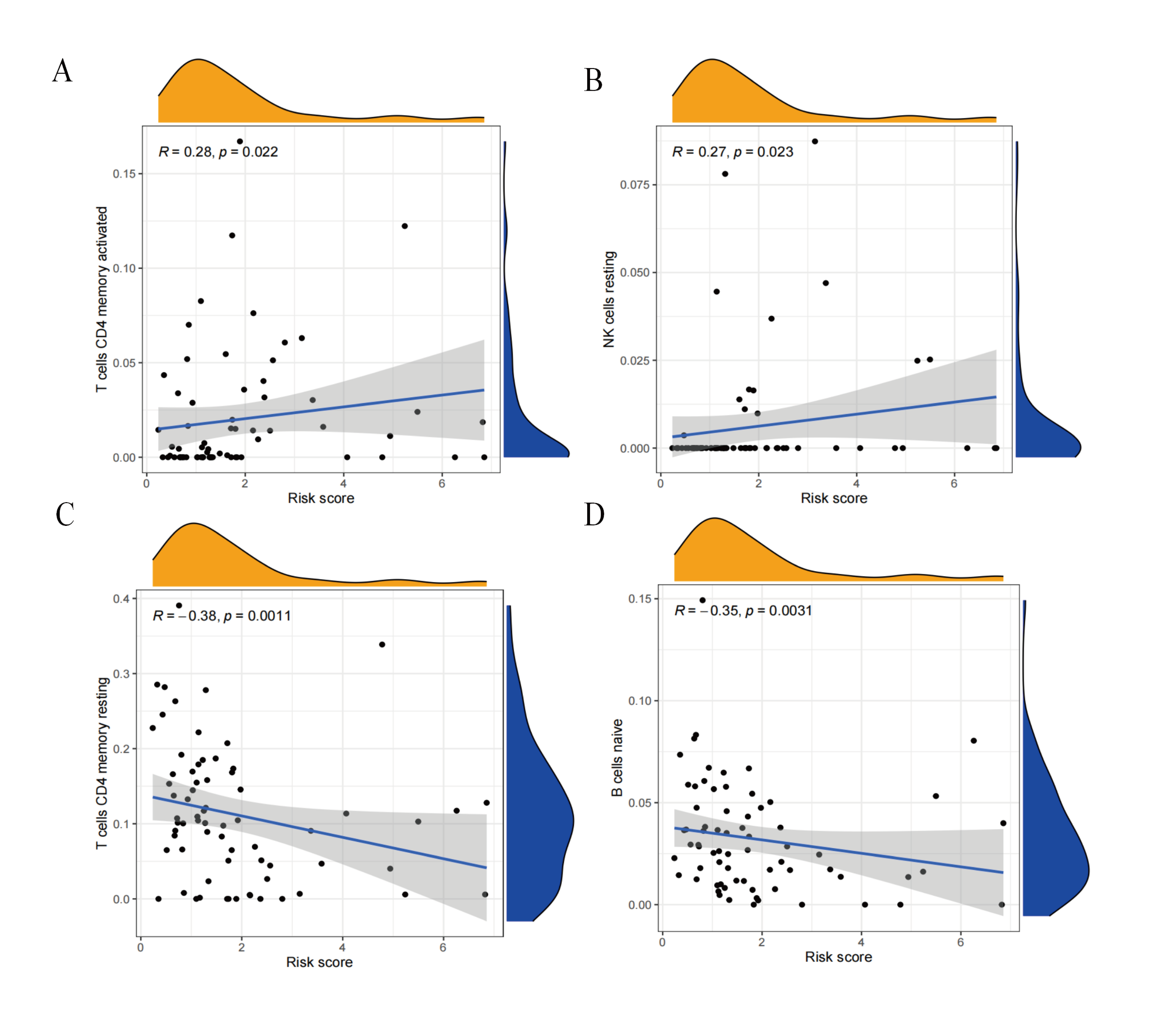

Supplement: Supplementary file 5 [file Image_5.tif]
