# Supplementary material for: Signature construction and molecular subtype identification based on cuproptosis-related genes to predict the prognosis and immune activity of patients with hepatocellular carcinoma
Source: Front Immunol. 2022 Sep 28;13:990790. doi: 10.3389/fimmu.2022.990790 (PMC9555242; doi:10.3389/fimmu.2022.990790)
Supplement: Supplementary file 6 [file Table_1.docx]

**Supplementary Table1. Primers target sequences.**

| name | sequences |
| --- | --- |
| **Primers for real-time PCR:** |  |
| TAF6 sense: | 5'-TGACATTGACTACGCCTTGAAG-3′ |
| TAF6 antisense: | 5'-TCCTCATAGAAGTAAAGCTCCCG-3′ |
| SPP2 sense: | 5'-CCGTATCTGTTTCGGGCATTC-3′ |
| SPP2 antisense: | 5'-GGACACATAGTAGTCCCTCTGCA-3′ |
| CFHR4 sense: | 5'-TGCGGTTTAAGCTCCATGACA-3′ |
| CFHR4 antisense: | 5'-CCCATCTTCACCACACACTATG-3′ |
| DNASE1L3 sense: | 5'-GATGAGTTGGTTGAGGTCTACAC-3′ |
| DNASE1L3 antisense: | 5'-CCGGCATTGAAGTCACCCAT-3′ |
| GAPDH sense: | 5'-AGAAGGCTGGGGCTCATTTG-3′ |
| GAPDH antisense: | 5'-AGGGGCCATCCACAGTCTTC-3′ |
| **The target sites of siRNA:** |  |
| si-TAF6#1 sense： | 5'-UCAUCUACCUGAUGCGUAU TT -3′ |
| si-TAF6#1 antisense： | 5'-AUACGCAUCAGGUAGAUGA TT-3′ |
| si-TAF6#2 sense： | 5'-CGAUGUUAUCAAGACUCUGAU TT -3′ |
| si-TAF6#2 antisense： | 5'-AUCAGAGUCUUGAUAACAUCG TT-3′ |
| si-TAF6#3 sense： | 5'-CCCCGGAUUCCCACACAUGCA TT-3′ |
| si-TAF6#3 antisense： | 5'-UGCAUGUGUGGGAAUCCGGGG TT-3′ |
| si-NC sense： | 5'-UUCUCCGAACGUGUCACGU TT-3′ |
| si-NC antisense： | 5'-ACGUGACACGUUCGGAGAA TT -3′ |
